# Pregnancy and delivery while receiving vagus nerve stimulation for the treatment of major depression: a case report

**DOI:** 10.1186/1744-859X-4-16

**Published:** 2005-09-16

**Authors:** Mustafa M Husain, Diane Stegman, Kenneth Trevino

**Affiliations:** 1 University of Texas Southwestern Medical Center at Dallas, 5323 Harry Hines Blvd, Dallas, Texas 75390-8898, USA

## Abstract

**Background:**

Depression during pregnancy can have significant health consequences for the mother and her infant. Antidepressant medications, which pass through the placenta, may increase the risk of low birth weight and preterm delivery. The use of selective serotonin reuptake inhibitors (SSRIs) during pregnancy may induce serotonergic symptoms in the infant after delivery. Antidepressant medications in breast milk may also be passed to an infant. Vagus nerve stimulation (VNS) therapy is an effective non-pharmacologic treatment for treatment-resistant depression (TRD), but little information exists regarding the use of VNS therapy during pregnancy.

**Case presentation:**

The patient began receiving VNS therapy for TRD in March 1999. The therapy was effective, producing substantial reductions in depressive symptoms and improvement of function. In 2002, the patient reported that she was pregnant. She continued receiving VNS therapy throughout her pregnancy, labor, and delivery, which enabled the sustained remission of her depression. The pregnancy was uneventful; a healthy daughter was delivered at full term.

**Conclusion:**

In this case, VNS therapy provided effective treatment for TRD during pregnancy and delivery. VNS was safe for the patient and her child.

## Background

A pregnant patient with major depression requires effective management of depressive symptoms for her own health and that of her child. Estimates of the prevalence of depression among pregnant women vary widely, ranging from 3.3% for major depression [1] to 20% for any type of depression [2]. Rates of depression may be as high as 51% in selected populations [3]. These rates compare with a 12-month worldwide prevalence of depression of 9.5% in women [4]. Among pregnant women with depression, many are untreated, sometimes discontinuing treatment for depression after becoming pregnant [1,5].

Depression during pregnancy can have many serious consequences. For the mother, depression is associated with an overall decline in general health, physical and social functioning, an increase in the experience of pain [3], and obstetric complications [6-8]. Depression in late pregnancy is associated with post-partum depression [2], while depression in early pregnancy increases the risk of preeclampsia, a major complication characterized by rapidly progressive hypertension with proteinuria, edema, or both [9]. For the infant, maternal depression during pregnancy was associated with admission to a neonatal intensive care unit [7] and with spontaneous preterm delivery in one study [10] but not in another [11].

Because of the importance of managing depression during pregnancy, numerous studies have examined the effects of antidepressant medications on fetal and infant development. Antidepressants and their metabolites pass through the placenta [12] and increase the risk of low birth weight [13,14] and preterm delivery [14,15]. Use of selective serotonin reuptake inhibitors (SSRIs) by mothers during pregnancy has been associated with substantially reduced levels of platelet serotonin in newborns [16], which may account for SSRI-induced serotonergic symptoms [17], serotonin withdrawal syndrome[18], tremulousness, reduced motor activity, heart rate variability [15], and blunted pain response [19,20]. Increased dosing of SSRIs may be required to maintain euthymia during later stages of pregnancy [21], which may exacerbate some effects. Antidepressants are transmitted to infants in breast milk, where they usually have no discernible clinical effect. However, in isolated reports, antidepressants in breast milk have been associated with reduced feeding, somnolence, reduced growth, and possible seizure [22]. Because both depression and its treatment with pharmacologic interventions may pose risks to the patient and her child, it is important to identify safe nonpharmacologic therapies for that may be used to treat major depressive episodes during pregnancy.

Vagus nerve stimulation (VNS) therapy has been evaluated for use in TRD [23-25]. A small pulse generator implanted subcutaneously in the left thoracic area delivers mild programmed pulses through an implanted lead to the left vagus nerve in the neck. Approved for the treatment of epilepsy since 1997, VNS therapy has been administered to more than 32,000 patients worldwide [26]. Several clinical studies have evaluated the use of adjunctive VNS therapy in chronic or recurrent TRD.

In a 3-month open-label pilot study of patients with chronic or recurrent TRD (bipolar or unipolar, defined by Diagnostic and Statistical Manual of Mental Disorders, 4th Edition (DSM-IV) criteria [27], patients receiving adjunctive VNS therapy exhibited statistically significant improvements in average scores on the Hamilton 28-Item Rating Scale for Depression (HRSD_28_), Montgomery Asberg Depressive Rating Scale (MADRS), Global Assessment of Function (GAF), and Clinical Global Impression – Severity (CGI-S) scales [24]. After a year of follow-up, adjunctive VNS therapy was associated with sustained symptomatic benefit and sustained or enhanced functional status [25].

Because pregnancy was a contraindication for enrollment in the VNS studies of patients with TRD, there have been no studies of the use of VNS therapy among pregnant patients. A report of eight pregnancies in patients receiving VNS therapy for pharmacoresistent epilepsy has been reported [28] concluded that VNS therapy does not prevent conception and is not associated with any adverse effects on the pregnancy or the neonate.

## Case presentation

The study from which this case report was derived was conducted in accordance with the ethical principles defined in the Declaration of Helsinki of the World Medical Association. The protocol was approved by the Institutional Review Boards (IRBs) of participating institutions, and each patient provided written informed consent.

The case study patient, a Caucasian woman aged 28 years with a DSM-IV diagnosis of unipolar depression, was enrolled in the acute and long-term phases of the pilot study of VNS therapy for TRD. At acute-phase study entry, she was noted to be obese and to have mild bronchoconstriction, as well as hypertension, sleep apnea, and arthritis in her knees, ankles, and feet. She reported that she had suffered from recurring depression for 10 years, confounded by obesity, despite pharmacologic treatment and psychotherapy. Her current depressive episode, which had begun 22 months before study enrollment, was found to be resistant to six different antidepressants (citalopram, sertraline, venlafaxine, paroxetine, bupropion, and clonazepam) and the atypical antipsychotic risperidone. In the year preceding enrollment in the study, she had been hospitalized twice for depression. The patient's baseline physical and clinical characteristics are summarized in Table 1.

**Table 1:** Baseline Physical and Clinical Characteristics of the Patient

**Characteristic**	
Age	28 years
Height	168 cm
Weight	160 kg
Heart rate (BPM)	106
Blood pressure	Systolic: 122 Diastolic: 88
Neurological examination	Sad/depressed affect; other parameters within normal limits
Clinical assessment	
HRSD_28_	49
MADRS	36
GAF	42
CGI-S	6

**cm: **centimeters; **kg: **kilograms; **BPM: **Beats per minute; **HRSD_28_: **Hamilton 28-Item Rating Scale for Depression; **MADRS: **Montgomery Asberg Depressive Rating Scale; **GAF: **Global Assessment of Function; **CGI-S: **Clinical Global Impression – Severity

**Figure 1:**
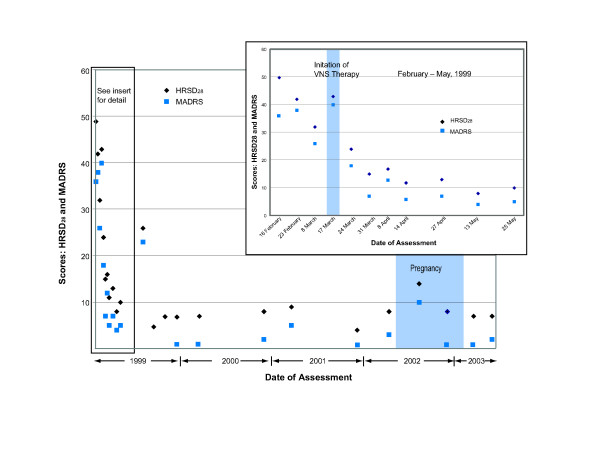
The patient experienced a substantial reduction in symptoms after receiving VNS therapy, as indicated by HRSD_28 _and MADRS scores. VNS therapy was initiated on March 17, 1999. The patient reported her pregnancy on May 30, 2002, and delivered a healthy child on January 24, 2003; remission of depression was sustained during the pregnancy.

The VNS therapy device and leads were surgically implanted on February 26, 1999. After recovery, the patient started receiving VNS therapy on March 17, 1999, with the initial stimulation parameters set as shown in Table 2. Substantial improvement was evident after 4 weeks, with depressive symptoms reduced and functioning improved as indicated by HRSD_28, _MADRS, CGI, and GAF scores (Figures 1 through 3). After 11 months, her HRSD_28 _score had decreased to 7 and her GAF score had reached 96. The patient's VNS output current was reduced to 0.25 milliamperes (mA) on August 31, 2001; other stimulation parameters were unchanged.

**Figure 2:**
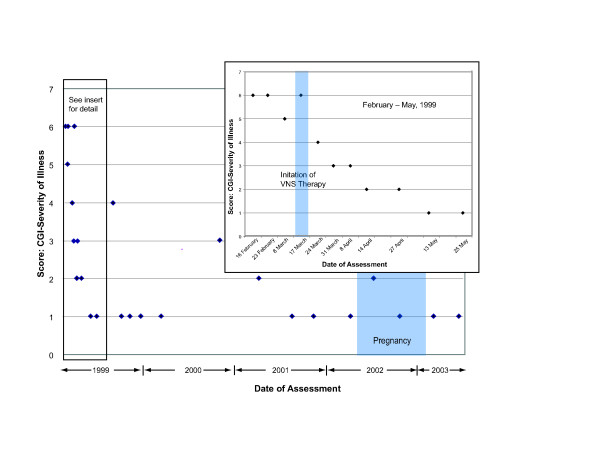
The patient's score on the CGI-S scale also indicated a substantial improvement after the initiation of VNS therapy. Pregnancy from May 30, 2002 to January 24, 2003 did not significantly affect the patient's CGI scores.

**Figure 3:**
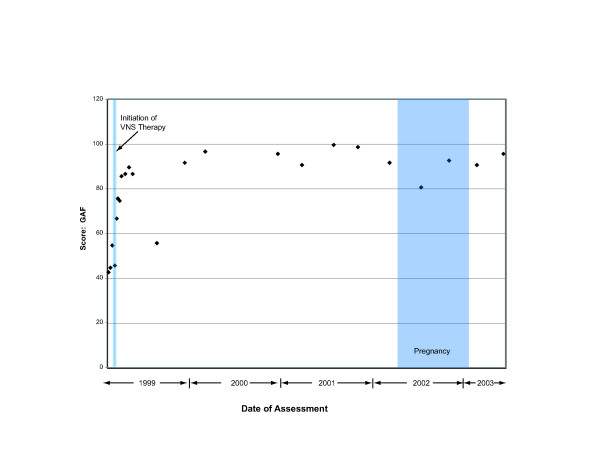
Improvement in functioning is demonstrated by increases in GAF scores, beginning shortly after the start of VNS therapy and continuing through her pregnancy and delivery.

**Table 2:** Vagus Nerve Stimulation Therapy Parameter Values

**Parameter**	**Value**		
	**March 17, 1999 (beginning of stimulation)**	**August 2001**	**During Pregnancy**

Output current	0.50 mA	0.25 mA	0.25 mA
Signal frequency	20 Hz	20 Hz	20 Hz
Pulse width	500 μsec	250 μsec	250 μsec
Signal ON time	30 sec	30 sec	30 sec
Signal OFF time	5 min	5 min	5 min

**mA: **milliamperes; **Hz: **Hertz; **μsec: **microseconds; **sec: **seconds

With her depression in remission, the patient underwent gastric bypass surgery for obesity on December 22, 2000. The pulse generator was turned off on December 21, 2000 in preparation for her surgery; VNS therapy was resumed on March 27, 2001. Twoyears after the surgery, she had lost approximately 55 kg.

On May 30, 2002, the patient reported that she was pregnant with her first child. She was informed that, while information was limited on the effects of VNS therapy during pregnancy, no safety issues were known that would affect the pregnancy. The patient decided to continue receiving VNS therapy during the pregnancy; no changes were made in stimulation parameters. In addition, she continued to receive citalopram 80 mg per day and bupropion 400 mg per day, after dosage reductions were considered and rejected by her physicians. She remained in clinical remission of depression throughout her pregnancy (Figures 1 through 3). In compliance with the clinical study protocol, the pregnancy was reported as a serious adverse event that was not related to VNS therapy.

After an uneventful gestation period and normal spontaneous vaginal delivery with epidural anesthesia, the patient delivered a healthy daughter at full term on January 24, 2003. The infant weighed 3.1 kg and was approximately 49 cm long.

VNS therapy was administered at the patient's normal settings throughout labor and delivery. Contingency plans had been made to discontinue stimulation if the patient had required a Caesarian section procedure in which electrocautery might be used. (To avoid damage to the pulse generator and leads, the manufacturer recommends that electrosurgery electrodes be placed as far as possible from the implant, out of the direct path of current flow. Confirmation of correct programmed function of the device after electrosurgery is also recommended.) However, the patient did not require a Caesarian section, and programmed VNS therapy was continued during labor and delivery.

The patient reported an episode of postpartum depression lasting 11 days after delivery. She attributed the depressive episode to difficulties in breast-feeding, and the episode resolved without specific treatment. The child, now aged approximately two years, exhibits normal age-appropriate development.

Another serious adverse event, which was not considered to be associated with VNS therapy, occurred after implant: an episode of thrombophlebitis that resolved with medical therapy. Mild adverse events that the study investigator considered possibly or definitely related to the implant procedure or to VNS therapy were one episode each of moderate leg pain, discomfort in the lower incisors during stimulation, dizziness that resulted in a fall, and surgical wound opening. The patient also experienced periodic hoarseness, a common side effect associated with VNS therapy that is considered tolerable by most patients. No adverse events associated with the VNS therapy occurred during pregnancy, labor, or delivery.

## Conclusion

Management of pregnancy in a woman with depression requires careful monitoring and treatment of depressive symptoms in addition to other aspects of the patient's condition. This patient, who had severe depression, experienced sustained remission of her TRD during pregnancy while receiving VNS therapy in combination with citalopram 80 mg per day and bupropion 400 mg per day. In this case, VNS therapy provided effective adjunctive treatment for the patient's depression during pregnancy and delivery; VNS was safe for the patient and her child.

## Competing interests

Mustafa Husain declares that, in the last five years, he has received research grants from Cyberonics, Inc. and is on the Cyberonics Speakers' Bureau. Cyberonics, Inc. is funding the development and article processing fees associated with this manuscript. Dr. Husain further declares that he does not own any stock in Cyberonics, Inc.

Diane Stegman declares that, in the last five years, she has received fees from Cyberonics, Inc. for her work as clinical study coordinator. Ms. Stegman further declares that she does not own any stock in Cyberonics, Inc.

Kenneth Trevino declares that he has no competing interests.

## Authors' contributions

MMH was the principal investigator of the VNS pilot study. DS was the study coordinator. KT assisted in data analysis and manuscript preparation.
